# Manipulability Inclusive Principle for Assistive Result Evaluation of Assistive Mechanism

**DOI:** 10.1155/2018/2767129

**Published:** 2018-09-24

**Authors:** Wenyuan Liang, Yong Yu

**Affiliations:** ^1^College of Engineering, Peking University, Beijing, China; ^2^National Research Center for Rehabilitation Technical Aids, Beijing, China; ^3^Graduate School of Science and Engineering, Kagoshima University, Kagoshima, Japan

## Abstract

In this paper, from the aspect of kinematics, we reveal the physical significance of assistance for the assistive mechanism. Then, Manipulability Inclusive Principle (MIP) is proposed to evaluate assistive mechanism's assistive feasibility and assistive effect through the manipulability comparison between the assisted limb and slave-active-assistive mechanism. The optimization based on MIP can make the assistive mechanism realize better kinematical performance and assistance. The design and optimization of the assistive mechanism should keep the assistive mechanism from interfering with human's movements in the expected workspace. More importantly, it should also keep the assistive mechanism not only have better kinematical performance on symmetry, isotropy, etc. but also be able to provide better assistance for human. The application on the human lower-limb straight-walking power-assisting mechanism shows that the design and optimization based on MIP can find out the assistive mechanism which satisfies assistive feasibility and realizes better assistive effect in the whole expected workspace.

## 1. Introduction

For the purpose of helping the elders and disabled, a power-assisting robot [1, 2] has been devised and defined as an active mechanical device that fits closely for the operator's body and works in concert with the operator's movements. In the last century, late 60s and early 70s, the Hardiman project [3], a large two-armed, bipedal exoskeletal system, operated at GE was controlled by using a master-slave system. The Belgrade exoskeleton [4] was a human-sized lower-extremity robot designed to help the paraplegics realize rehabilitation. The Lokomat system [5] is a walker system for standing assistance [6].

In recent years, there are more assistive devices appeared. The wearable power-assisting system also becomes an attractive method of power assisting since it can be adapted to a wide range of applications. For example, Sarcos Exoskeleton [7], Wearable Power Suit [8], and Hybrid Assistive Limb (HAL) [9] are full-body assistive robots; MGA Exoskeleton [10], SUEFUL-6 [11], and CADEN-7 [12] focus on upper-limb assistance; Berkeley Lower Extremity Exoskeleton (BLEEX) [13], MIT Exoskeleton [14], RoboKnee [15], Human Universal Load Carrier (HULC) [16], Wearable Walking Helper-KH [17], and Bodyweight Support System [18] are the assistive robots which are used for lower-limb assistance. On the power-assisting robot, we also have made some research studies, such as PAWL [19, 20] which is designed for human lower-limb assistance, upper-limb assistive mechanism [21], and a parallel assisting mechanism for hip joint 3-DOF power assisting [22].

In this paper, we focus on the wearable assistive mechanism which belongs to the following type: the human is acting actively, and simultaneously, the assistive robot works actively to provide assistance with matching human's moving intention, where [7–22] all belong to this type. When this kind of assistive mechanism is mounted to the human body, the assistive mechanism and human are considered as a parallel mechanism, where the active joints of this parallel mechanism are divided into two types: master-active joints and slave-active joints. For this parallel mechanism, it has several kinds of problems, such as selecting slave-active joints, confirming types of joints, configurations and sizes, motion interference, sensors, and control. All these problems will have the influence on the final assistance, including assistive feasibility and assistive effect.

In order to realize better assistance, these problems should be partly considered during the assistive mechanism design and optimization, where the rest is determined by the sensors and control. Generally, for the mechanism design and optimization [23, 24], researchers have considered it from these aspects: workspace, symmetry, isotropy, and so on. For this parallel mechanism which consists of the assistive mechanism and human, by considering that the assistive mechanism's own special characteristic is that the assistive mechanism and human cooperate together to realize assistance; the design and optimization should keep the assistive mechanism from interfering with human's movements in the expected workspace. And especially, the design and optimization should also keep the assistive mechanism not only have better kinematical performance on symmetry, isotropy etc., but also be able to provide better assistance for human. In recent research studies, the research in [30] uses the Jacobian matrices to analyze the assistive isotropy and assistive efficiency; researchers in [31] use the global performance index to represent the dexterity, and the manipulability ellipsoids for the 4-DOF assistive robot is obtained in [32]. However, the research studies shown in [30–32] have not considered the influence of the motions of the human limb. For example, the problem of assistive feasibility should compare the manipulability between the assistive mechanism and the human limb. Additionally, compared to [30–32], this paper will show more systemic research on optimization problem including assistive feasibility and assistive efficiency.

In this paper, from the aspect of kinematics, we propose Manipulability Inclusive Principle (MIP), which is derived from the physical significance of assistance and based on robotics manipulability theory [25–27], to consider the problems during assistive mechanism's design and optimization. The prototype of MIP first appeared in our pre-study [22], used on hip joint 3-DOF assistive mechanism's design and optimization. The MIP is related to actuators' maximum velocities, assistive mechanism's fixed and connected locations, mechanical structure, configuration, types of joints, sizes, and so on. The optimization algorithm based on MIP is helpful for designing and optimizing the assistive mechanism in terms of kinematics, finding out the structure which can realize better kinematical performance and assistance. In theory, the MIP is also applicable for many kinds of power-assisting robots which belong to the type where human is acting actively, and the assistive mechanism is matching human's moving intention.

The MIP has been partially discussed in the pre-work [22, 28, 29], where the content in this paper is the supplement for the pre-work. For the sake of discussing, an example of lower-limb assistive robot is used in this paper. This paper is organized as follows: some definitions and the problems are shown in Section 2.  Section 3 presents the definition of MIP. In Section 4, with some examples and analysis, the MIP is proved to be able to evaluate lower-limb assistive mechanism's design and optimization. Conclusions are presented in Section 5.

## 2. Slave-Active-Assistive Mechanism

**Figure 1:**
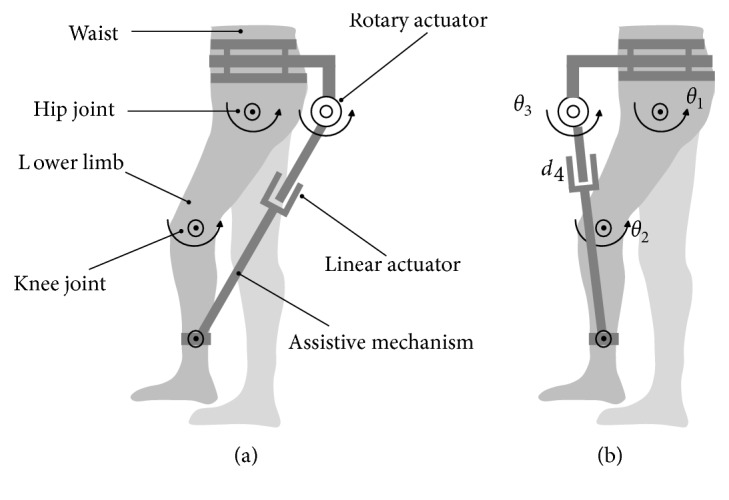
Lower-limb assistive mechanism for straight-walking assistance.

As well known, for the human lower limb, hip joint has 3 DOFs, and the knee joint is a 1-DOF joint. Similar to the lower-limb straight-walking assistive robots in [16, 17], in this paper, the lower-limb straight-walking assistive mechanism also only assists hip joint's and knee joint's flexion/extension motions, where the assistive mechanism includes a revolute joint and a prismatic joint as shown in Figure 1. This assistive mechanism is fixed to the human waist and connected to the calf with a revolute joint. For example, research studies in [16, 17] place the assistive robots just as shown in Figure 1(a), where the assistive mechanism is in the behind of the human body; Figure 1(b) shows the model where the assistive mechanism is in front of the human body.

For the sake of discussing and understanding, before discussing the problems for this lower-limb assistive mechanism, first of all, we propose some important and useful definitions for power assisting as follows.

### 2.1. Master-Active Joint and Slave-Active Joint

When the assistive mechanism is mounted to the human body, the assistive mechanism and human can be considered as a parallel mechanism. In general, the joints of parallel mechanism are divided into two types: active joints and passive joints, where the joint with driven source is defined as the active joint and the joint without driven source is defined as the passive joint. If too many joints are considered as active joints, it will lead to an interference between these active joints, and if too few joints are considered as active joints, this parallel mechanism cannot be driven well. Hence for a parallel mechanism, the number of active joints should be equal to the number of the DOFs of the parallel mechanism.

However, during assisting, both human's joints and the actuators installed on the assistive mechanism are with the driven source, providing power on human motions, where the assistance cannot be realized without these actuators. Hence, in this parallel mechanism which consists of the assistive mechanism and human, there is a problem that the number of active joints is not equal to the number of the DOFs of the parallel mechanism, where it seems that this problem will lead to interference between the human and assistive mechanism. This problem can be considered as follows:

For a power-assisting robot, the human is at the master level and generating the moving intention, and the assistive mechanism is at the slave level to match human's moving intention, where the assistive mechanism itself cannot generate moving intention. Therefore, for the active joints in this parallel mechanism, human's joints are acting actively and generating moving intention; therefore, human's joints are defined as master-active joints; the assistive mechanism is matching human's moving intention and then working actively to provide necessary assistance; therefore, assistive mechanism's joints with driven sources are defined as slave-active joints.

The slave-active joints drive the parallel mechanism to match human's movements; therefore, the number of slave-active joints should be equal to the number of the DOFs of this parallel mechanism. In addition, when selecting different assistive mechanisms' joints as slave-active joints, the assistive results will be different.

In this paper, the parallel mechanism, which consists of the lower limb and assistive mechanism, has 2 DOFs just the same as the lower limb; therefore, it is necessary to use 2 slave-active joints to assist the lower limb. By considering the actuators' installed locations and weight, the revolute joint (with rotary actuator) and prismatic joint (with linear actuator) are selected as slave-active joints just as shown in Figure 1. The left revolute joint connecting the assistive mechanism and lower limb is passive joint.

### 2.2. Assisted Limb and Slave-Active-Assistive Mechanism

For power assisting, the active joints are divided into master-active joints and slave-active joints. In other words, human and master-active joints are at the master level, and assistive mechanism and slave-active joints are at the slave level. For these two levels, we can have another two definitions.

The assisted limb represents the part of the human body which is to be assisted and only driven by the master-active joints. In this paper, the assisted limb is the human lower limb, and the master-active joints are composed of the hip joint and the knee joint.

Slave-Active-Assistive Mechanism (SAAM), a parallel mechanism consisting of the assistive mechanism and assisted limb, is only driven by the slave-active joints, where in this parallel mechanism, master-active joints are also considered as passive joints. The reason for proposing the definition of SAAM is that since the assistive mechanism is finally mounted to the assisted limb, discussing assistive mechanism's assistive performance should combine with the assisted limb.

The discussions and comparisons in this paper are based on these two definitions: the assisted limb representing lower limb's performance and SAAM representing assistive mechanism's performance. Actually, the assisted limb and SAAM are acting simultaneously as a whole during assisting. By comparing their kinematical performances, assistive mechanism's assistive results can be judged in terms of kinematics.

### 2.3. Problems: Assistive Feasibility and Assistive Effect

Combined with the definitions above, the problems for lower-limb assistive mechanism's design and optimization are discussed as follows. As shown in Figure 1, the assistive mechanism is mounted to the assisted limb with different locations, and the assistive results may be different. Besides, the parameters such as actuators' maximum velocities, mechanical structure, types of joints and actuators, and sizes would also have the influence on the assistive results. In general, with different parameters, assistive mechanism's assistive results are different.

Therefore, the purpose in this paper is optimizing lower-limb assistive mechanism's parameters to realize better assistive results, where in this paper, the discussions focus on the parameters of actuators' maximum velocities and assistive mechanism's fixed location.

Realizing better assistive results includes a double meaning: first, the assistive mechanism must satisfy assistive feasibility; then further, it should realize better assistive effect. Assistive feasibility means that the assistive mechanism is able to provide necessary assistance on the expected motions, where in this paper, the expected motions are lower limb's straight-walking motions. Assistive effect is used to evaluate the final assistance, where the assistive effect includes three aspects such as assistive efficiency, assistive ability, and assistive isotropy.

In order to realize better assistive results, during lower-limb assistive mechanism's design and optimization, we should consider these two problems: assistive feasibility and assistive effect, where these two problems also exist in many other assistive mechanisms.

#### 2.3.1. Problem of Assistive Feasibility

On the problem of assistive feasibility, it does have three concerns.

First, the corresponding type of SAAM end-effector's DOF should be the same as the lower limb. Second, SAAM end-effector's DOF space and lower-limb end-effector's DOF space should be in the same space. Avoiding interfering lower limb's movements, these two concerns ensure that when the slave-active joints are without actuators, the assistive mechanism can move with lower limb's movements.

Third, the assistive mechanism, where the slave-active joints are driven by the actuators, should be able to catch the lower limb's movements. As said before, during the assisting process, the assistive mechanism is acting actively to match lower limb's moving intention. If the assistive mechanism cannot catch lower limb's movements, the assistive mechanism still cannot assist even if their end-effectors' DOF spaces are in the same space.

Thus, satisfying these three concerns ensures that the assistive mechanism is able to shadow lower limb's movements and provide necessary assistance for the lower limb.

#### 2.3.2. Problem of Assistive Effect

Another problem is the assistive effect. When the assistive mechanism satisfies assistive feasibility, SAAM end-effector's and lower-limb end-effector's DOF spaces are in the same space. However, since the DOF direction and magnitude may be different, the assistive effect may be different.

In order to realize better assistive effect, it is necessary to discuss its included three aspects further. Assistive ability means the assisting power which the assistive mechanism owns. Assistive efficiency shows that how much assistive mechanism's assisting power can apply on assisted limb's motions. And the similarity of assistive ability on each DOF is considered as assistive isotropy.

Assistive mechanism's assistive feasibility and assistive effect are related to the parameters, such as actuators' maximum velocities, assistive mechanism's fixed location, and so on. Therefore, proposing an evaluation criterion which can consider the assistive feasibility and assistive effect is of great significance. Then the evaluation criterion can be used for assistive mechanism's design and optimization.

In this paper, Manipulability Inclusive Principle (MIP) is proposed to consider the problems of assistive feasibility and assistive effect. When SAAM's manipulability ellipsoid and lower limb's manipulability ellipsoid are in the same space, their end-effector's DOF spaces are also in the same space. Then, while SAAM's manipulability ellipsoid can cover lower limb's manipulability ellipsoid, the assistive mechanism satisfies assistive feasibility to provide assistance on all the DOFs. The assistive effect is considered from the differences of principle axes' directions and volume and axes' magnitude between SAAM's and lower limb's manipulability ellipsoids.

Better assistance means the assistive mechanism can use less power to realize more assistance. In order to realize better assistance in the whole expected workspace, optimization based on MIP is helpful for optimizing assistive mechanism in terms of kinematics.

## 3. Manipulability Inclusive Principle

In order to consider the problems of assistive feasibility and assistive effect, from the aspect of kinematics, Manipulability Inclusive Principle (MIP) is proposed to evaluate lower-limb assistive mechanism's design and optimization to realize better assistance. In the following, we will show the definition of the MIP evaluation criterion which is derived from the physical significance of assistance.

### 3.1. Physical Significance of Assistance for Assistive Mechanism

**Figure 2:**
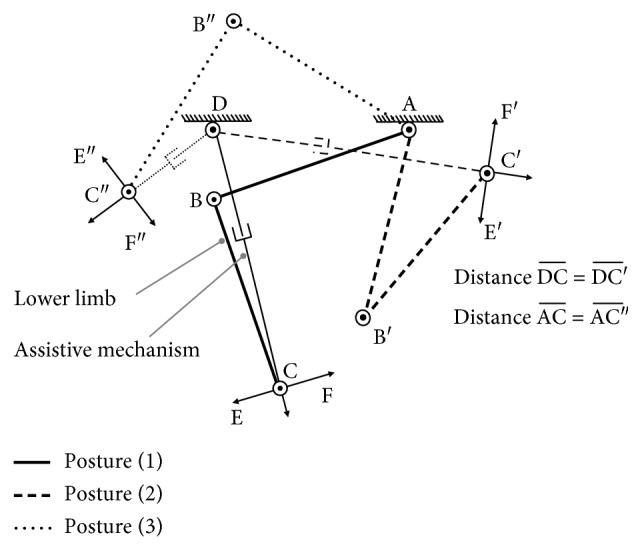
Lower limb with assistive mechanism at different postures.

Let us see an example, where the lower limb and assistive mechanism in Figure 1(b) is simply represented as an RR-R-PR parallel mechanism ABCD shown in Figure 2. In this parallel mechanism, lower limb ABC and assistive mechanism DC are connected at C; assistive mechanism's fixed location is D, and connected location is C. As the discussion for the definitions of master-active joints and slave-active joints before, here master-active joints A and B separately represent hip joint and knee joint; rotary actuator and linear actuator are adopted as the two slave-active joints' driven sources; the left revolute joint C is a passive joint.

Figure 2 shows that the lower limb and assistive mechanism are at three different postures, where on the distance, there are $\bar{\text{DC}}={\bar{\text{DC}}}^{\prime }$ and $\bar{\text{AC}}={\bar{\text{AC}}}^{\prime \prime }$. In this example, we focus on the assistance on the directions which are vertical to the direction from assistive mechanism's fixed location D to end-effector C, such as $\vec{\text{EF}}$, $\vec{{\text{E}}^{\prime }{\text{F}}^{\prime }}$, $\vec{{\text{E}}^{\prime \prime }{\text{F}}^{\prime \prime }}$, and so on. Assume that each joint for the lower limb and assistive mechanism separately has its own maximum velocity; at posture (1), when lower limb's end-effector C moves on direction $\vec{\text{EF}}$ at maximum velocity, the slave-active joint D also just reaches its maximum velocity to make the assistive mechanism shadow lower limb's movement to provide certain assistance on this direction.

Compared to the results on the direction $\vec{\text{EF}}$ of posture (1), intuitively at posture (2), lower-limb end-effector's maximum velocity decreases on direction $\vec{{\text{E}}^{\prime }{\text{F}}^{\prime }}$, so that slave-active joint D is able to use lower velocity to shadow lower limb's movement in this direction, where in this case assistive mechanism also can assist the lower limb much better on this direction; at posture (3), the distance between D and C^″^ becomes much shorter, leading that even slave-active joint D uses its maximum velocity, assistive mechanism still cannot shadow and assist the lower limb on direction $\vec{{\text{E}}^{\prime }{\text{F}}^{\prime }}$.

The above is an intuitive description; however, it reveals the physical significance of assistance described as following. After the assistive mechanism is mounted to the assisted limb, when lower limb's end-effector reaches maximum velocity on a certain direction, in this case, that slave-active joints' velocities do not exceed their own maximum velocities ensures the assistive mechanism is able to shadow the lower limb's movement to assist lower limb in this direction. And further, that slave-active joints can use lower velocities to shadow lower limb's movement means assistive mechanism can provide better assistance in this direction.

In other words, combined with the definitions of the assisted limb and SAAM, the physical significance of assistance also can be described as following. While SAAM end-effector's maximum velocity is not lower than assisted-limb end-effector's velocity on a certain direction, it means during the assisted limb's movements even assisted limb's end-effector reaches its maximum velocity, and slave-active joints' velocities are still lower than or equal to their own maximum velocities. Then, in this case, it ensures the assistive mechanism is able to shadow assisted limb's movement to assist on this direction. Therefore, we can compare assisted limb end-effector's and SAAM end-effector's maximum velocities to evaluate the assistive result in a certain direction.

In order to evaluate the assistive results on all the directions, comparing their end-effectors' maximum velocities one direction by one direction is a feasible method. For example, in this example, through comparing, at posture (2), the assistive mechanism can shadow lower limb's movements on all the directions; at posture (3), the assistive mechanism can shadow lower limb's movements on part of the whole directions.

However, the aforementioned method will incur a huge cost and will be ineffective to be applicable in practices, whereas another method based on the manipulability concept is adopted to consider evaluating assistive results on all the directions. Manipulability is a useful means of quantifying end-effector's velocity on each DOF, where it includes two ideas: manipulability direction and manipulability magnitude of each DOF. As a mathematical tool, velocity manipulability ellipsoid is used to describe end-effector's velocities on all the directions: along the manipulability direction of the major axis of the manipulability ellipsoid, the end-effector can move at large velocity, while along the manipulability direction of the minor axis, small end-effector velocities are obtained. In this paper, the velocity manipulability ellipsoid is simply called as manipulability ellipsoid.

Therefore, this adopted method, MIP, is based on comparing assisted limb's manipulability and SAAM's manipulability to consider the two problems of assistive feasibility and assistive effect, whereas in this paper, the manipulability only represents end-effector's manipulability. Then, the definition of MIP can be derived from the physical significance of assistance as follows.

### 3.2. Definition of Manipulability Inclusive Principle

Based on the manipulability concept, during the design and optimization for the assistive mechanism which belongs to the type where human is acting actively and simultaneously the assistive mechanism is matching human's moving intention just as shown in Section 1, the Manipulability Inclusive Principle (MIP) evaluation criterion is proposed to evaluate the assistive mechanism's assistive feasibility and assistive effect in terms of kinematics. Assuming that SAAM and the assisted limb are both at the same posture, for the same end-effector, the definition of MIP can be set out as follows:

- On the assistive feasibility, if assisted limb's manipulability ellipsoid is whole inclusive by SAAM's manipulability ellipsoid, the assistive mechanism can shadow the assisted limb's movements well to satisfy assistive feasibility and be used to provide power assisting on all the DOFs.
- On the assistive effect, if the corresponding manipulability directions of SAAM align with assisted limb's better, the assistive efficiency is higher; if the manipulability magnitude of SAAM is larger, the assistive mechanism owns more assistive ability; if the manipulability magnitude of SAAM on each DOF is closer, the assistive isotropy is better.

Assisting all the DOFs also means assisting all the directions, and manipulability directions represent the directions of the manipulability ellipsoid's principle axes.

As for the definition of MIP, from the macroscopic perspective, three inclusive cases can be derived from the assistive feasibility: whole inclusive case, part inclusive case, and no inclusive case. The whole inclusive case ensures that the assistive mechanism satisfies assistive feasibility to shadow assisted limb's movements well to assist all the DOFs, while the part inclusive case or no inclusive case means the assistive mechanism cannot assist assisted limb's movements in some directions or at all.

From the microscopic perspective, the inclusive results will have the influence on the assistive effect. The assisted limb's manipulability directions represent assisted limb's most needed assisted directions. The SAAM's manipulability directions are the directions where SAAM can provide its largest assistance. Therefore, the SAAM's manipulability directions align with assisted limb's better, the assistive efficiency is higher. In practice, as their directions cannot align completely, the other two factors, assistive ability and assistive isotropy, are needed to be considered. Too small assistive ability will lead to that SAAM's manipulability ellipsoid cannot cover assisted limb's, while too large assistive ability means a waste. Hence, during design and optimization for the assistive mechanism, we should select suitable actuators for the assistive mechanism to make the assistive mechanism satisfy assistive feasibility. With the limited assistive ability, we should keep the assistive mechanism have close assistive ability in each direction or DOF. Then, this assistive mechanism can realize better assistive effect.

From assisted limb's manipulability and SAAM's manipulability, the following can be obtained: Manipulability Ellipsoid of Assisted Limb (ME-al) and Manipulability Directions of Assisted Limb (MD-al) where in this case, the assisted limb is without assistive mechanism, the Manipulability Ellipsoid of SAAM (ME-saam) and Manipulability Directions of SAAM (MD-saam) where the SAAM is only driven by the slave-active joints. Then, EV-al and EV-saam separately represent end-effector's velocity of assisted limb and end-effector's velocity of SAAM, respectively.

## 4. MIP Studies with the Example of Lower-Limb Assistance

With Manipulability Inclusive Principle (MIP), it is necessary to validate that MIP is applicable on the lower-limb assistive mechanism, and then, the design and optimization for the lower-limb assistive mechanism can be operated with MIP. Therefore, it is needed to validate that MIP is applicable to evaluate assistive feasibility for lower-limb assistive mechanism and then, analyze and discuss the factors which will have the influence on assistive feasibility and assistive effect.

In this section, the validation, analysis, and discussion all are based on the manipulability. Manipulability can be obtained through singular value decomposition of the kinematical Jacobian. Associated with the normalized kinematical Jacobian *J*, there is a mathematical form:

(1) $$\begin{array}{c} J=U\cdot S\cdot V. \end{array}$$

Consequently, the above equation can generate a velocity manipulability ellipsoid which represents end-effector's moving velocities on all the directions. The principal axes of the ellipsoid, representing the manipulability directions of all the DOFs, are aligned with the matrix *U*, and the length of each principal axis which is equal to manipulability magnitude of each DOF is diagonal with matrix *S*. With the computing results, we can draw manipulability ellipsoids locating their centers at the locations of the end-effector in the workspace so that the globe characteristics of the end-effector's velocities can be represented in the whole.

In this section, the Jacobian and manipulability for assisted limb and SAAM are built at first. With the results generated from the Jacobian and manipulability, the validation on evaluating assistive feasibility for MIP is operated with some examples. Then, through the analysis between ME-al and ME-saam, an inclusive judgement algorithm is proposed to evaluate the inclusive cases. By learning about all kinds of inclusive cases, we can find out which inclusive case is our need. At last with the analysis and discussion on the influence factors of MIP, we will find the ways to improve assistance.

### 4.1. Jacobian and Manipulability for Lower Limb and SAAM

**Figure 3:**
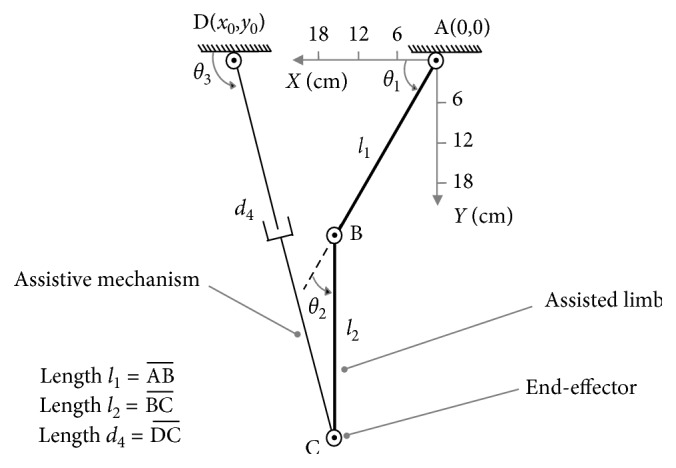
RR-R-PR mechanical configuration.

As shown in Figure 3, the reference coordinate A − *XY* is located at the hip joint. In this parallel mechanism ABCD, *l*_1_ represents the length between hip joint A and knee joint B, and *l*_2_ is the length from knee joint B to assistive mechanism's connected location C; *θ*_1_ and *θ*_2_ are the angular variables for the hip joint and knee joint on the motions of flexion/extension; in this assistive mechanism, *θ*_3_ represents the angular variable for revolute joint C, and *d*_4_ represents the variable of the length of the prismatic joint. In this paper, the unit of the length is (cm), and the unit for the angular variable is (rad); hence, the unit of linear velocity is (cm/s), and the unit of angular velocity is (rad/s). Therefore, the Jacobian for the lower limb and SAAM can be built as follows.

For lower limb, master-active joints A and B can determine the position of end-effector C, where *f*_1_ represents its *X*-position component, and *f*_2_ is the *X*-position component. The expressions for *f*_1_ and *f*_2_ are as follows:

(2) $$\begin{array}{c} f_{1}=l_{1}\cos\theta_{1}+l_{2}\cos\left(\theta_{1}+\theta_{2}\right), \end{array}$$

(3) $$\begin{array}{c} f_{2}=l_{1}\sin\theta_{1}+l_{2}\sin\left(\theta_{1}+\theta_{2}\right). \end{array}$$

For the assistive mechanism end-effector's position C, *f*_3_ and *f*_4_ separately represents its *X*-position component and *Y*-position component,

(4) $$\begin{array}{c} f_{3}=x_{0}+d_{4}\cos\theta_{3}, \end{array}$$

(5) $$\begin{array}{c} f_{4}=y_{0}+d_{4}\sin\theta_{3}, \end{array}$$

where (*x*_0_, *y*_0_) represents assistive mechanism's fixed location D. While the assistive mechanism is mounted to the lower limb, there are *x*_C_=*f*_1_=*f*_3_ and *y*_C_=*f*_2_=*f*_4_, where (*x*_C_, *y*_C_) represents the position of C.

For the lower limb which is driven by the master-active joints A and B, combined with Equations (2) and (3), the Jacobian is

(6) $$\begin{array}{c} {\left[\begin{array}{cc} {\dot{x}}_{\text{C}} & {\dot{y}}_{\text{C}} \end{array}\right]}^{T}=J_{01}\cdot {\left[\begin{array}{cc} {\dot{\theta }}_{1} & {\dot{\theta }}_{2} \end{array}\right]}^{T}, \end{array}$$

where [*∗*]^*T*^ is the transpose of matrix [*∗*]. In Equation (6), *J*_01_ is

(7) $$\begin{array}{c} J_{01}=\left[\begin{array}{cc} g_{11} & g_{12}\\ g_{21} & g_{22} \end{array}\right], \end{array}$$

where *g*_11_=∂*f*_1_/∂*θ*_1_, *g*_12_=∂*f*_1_/∂*θ*_2_, *g*_21_=∂*f*_2_/∂*θ*_1_, and *g*_22_=∂*f*_2_/∂*θ*_2_.

The Jacobian of SAAM is about the velocity relation between end-effector C and slave-active joints in the parallel mechanism ABCD; therefore, the Jacobian of SAAM has the following mathematical form:

(8) $$\begin{array}{c} {\left[\begin{array}{cc} {\dot{x}}_{\text{C}} & {\dot{y}}_{\text{C}} \end{array}\right]}^{T}=J_{02}\cdot {\left[\begin{array}{cc} {\dot{\theta }}_{3} & {\dot{d}}_{4} \end{array}\right]}^{T}. \end{array}$$

Then, it is needed to find out the expression of *J*_02_. As C connects the lower limb and assistive mechanism, the velocity of SAAM's end-effector C is related to the lower limb and assistive mechanism. Lower limb's Jacobian is expressed as Equation (6), and combined with Equations (4) and (5), the Jacobian of the assistive mechanism is

(9) $$\begin{array}{c} {\left[\begin{array}{cc} {\dot{x}}_{\text{C}} & {\dot{y}}_{\text{C}} \end{array}\right]}^{T}=J_{03}\cdot {\left[\begin{array}{cc} {\dot{\theta }}_{3} & {\dot{d}}_{4} \end{array}\right]}^{T},\\ J_{03}=\left[\begin{array}{cc} g_{31} & g_{32}\\ g_{41} & g_{42} \end{array}\right], \end{array}$$

where *g*_31_=∂*f*_3_/∂*θ*_3_, *g*_32_=∂*f*_3_/∂*θ*_4_, *g*_41_=∂*f*_4_/∂*θ*_3_, and *g*_42_=∂*f*_4_/∂*θ*_4_. When the assistive mechanism is mounted to the lower limb, there is

(10) $$\begin{array}{c} J_{01}\cdot {\left[\begin{array}{cc} {\dot{\theta }}_{1} & {\dot{\theta }}_{2} \end{array}\right]}^{T}=J_{03}\cdot {\left[\begin{array}{cc} {\dot{\theta }}_{3} & {\dot{d}}_{4} \end{array}\right]}^{T}. \end{array}$$

Therefore, the SAAM which is only driven by the slave-active joints, its Jacobian is

(11) $$\begin{array}{c} {\left[\begin{array}{cc} {\dot{x}}_{\text{C}} & {\dot{y}}_{\text{C}} \end{array}\right]}^{T}=\frac{\left(J_{01}\cdot {\left[\begin{array}{cc} {\dot{\theta }}_{1} & {\dot{\theta }}_{2} \end{array}\right]}^{T}+J_{03}\cdot {\left[\begin{array}{cc} {\dot{\theta }}_{3} & {\dot{d}}_{4} \end{array}\right]}^{T}\right)}{2}\\ \;\;=\frac{\left(J_{01}\cdot J_{01}^{+}\cdot J_{03}+J_{03}\right)}{2\cdot {\left[\begin{array}{cc} {\dot{\theta }}_{3} & {\dot{d}}_{4} \end{array}\right]}^{T}}, \end{array}$$

where [*∗*]^+^ represents the pseudo-inverse of [*∗*], *J*_02_=(*J*_01_ · *J*_01_^+^ · *J*_03_+*J*_03_)/2. When assisted limb is not at the singular postures, the Jacobian for SAAM becomes

(12) $$\begin{array}{c} {\left[\begin{array}{cc} {\dot{x}}_{\text{C}} & {\dot{y}}_{\text{C}} \end{array}\right]}^{T}=\frac{\left(J_{01}\cdot J_{01}^{-1}\cdot J_{03}{\left[\begin{array}{cc} {\dot{\theta }}_{3} & {\dot{d}}_{4} \end{array}\right]}^{T}+J_{03}\cdot {\left[\begin{array}{cc} {\dot{\theta }}_{3} & {\dot{d}}_{4} \end{array}\right]}^{T}\right)}{2}\\ =J_{03}\cdot {\left[\begin{array}{cc} {\dot{\theta }}_{3} & {\dot{d}}_{4} \end{array}\right]}^{T}, \end{array}$$

where [*∗*]^−1^ represents the inverse matrix of [*∗*], *J*_02_=*J*_03_. In this case, the Jacobian for SAAM and assistive mechanism are the same.

Then for assisted limb's and SAAM's separate Jacobian shown in Equations (6) and (8), the inputs are needed to be normalized [25] with their maximum input velocities, and the outputs are also needed to be normalized by limiting them with the maximum expected output velocities, where usually the maximum expected output velocities are assisted limb end-effector's maximum moving velocities. After normalization, there are

(13) $$\begin{array}{c} {\left[\begin{array}{cc} \frac{{\dot{x}}_{\text{C}}}{{\left({\dot{x}}_{\text{C}}\right)}_{\max}} & \frac{{\dot{y}}_{\text{C}}}{{\left({\dot{y}}_{\text{C}}\right)}_{\max}} \end{array}\right]}^{T}=A_{03}^{-1}\cdot J_{01}\cdot A_{01}\cdot {\left[\begin{array}{cc} \frac{{\dot{\theta }}_{1}}{{\left({\dot{\theta }}_{1}\right)}_{\max}} & \frac{{\dot{\theta }}_{2}}{{\left({\dot{\theta }}_{2}\right)}_{\max}} \end{array}\right]}^{T}, \end{array}$$

(14) $$\begin{array}{c} {\left[\begin{array}{cc} \frac{{\dot{x}}_{\text{C}}}{{\left({\dot{x}}_{\text{C}}\right)}_{\max}} & \frac{{\dot{y}}_{\text{C}}}{{\left({\dot{y}}_{\text{C}}\right)}_{\max}} \end{array}\right]}^{T}=A_{03}^{-1}\cdot J_{02}\cdot A_{02}\cdot {\left[\begin{array}{cc} \frac{{\dot{\theta }}_{3}}{{\left({\dot{\theta }}_{3}\right)}_{\max}} & \frac{{\dot{d}}_{4}}{{\left({\dot{d}}_{4}\right)}_{\max}} \end{array}\right]}^{T}, \end{array}$$

where ${\left({\dot{\theta }}_{1}\right)}_{\max}$, ${\left({\dot{\theta }}_{2}\right)}_{\max}$, ${\left({\dot{\theta }}_{3}\right)}_{\max}$, and ${\left({\dot{d}}_{4}\right)}_{\max}$ are the maximum velocities of master-active joints and slave-active joints, and ${\left({\dot{x}}_{\text{C}}\right)}_{\max}$, ${\left({\dot{y}}_{\text{C}}\right)}_{\max}$ are end-effector's maximum expected output velocities. Matrices *A*_01_, *A*_02_, and *A*_03_ are defined as

(15) $$\begin{array}{c} A_{01}=\mathrm{diag}\left({\left({\dot{\theta }}_{1}\right)}_{\max},{\left({\dot{\theta }}_{2}\right)}_{\max}\right),\\ A_{02}=\mathrm{diag}\left({\left({\dot{\theta }}_{3}\right)}_{\max},{\left({\dot{d}}_{4}\right)}_{\max}\right),\\ A_{03}=\mathrm{diag}\left({\left({\dot{x}}_{\text{C}}\right)}_{\max},{\left({\dot{y}}_{\text{C}}\right)}_{\max}\right), \end{array}$$

where diag(·) represents a diagonal matrix. In Equations (13) and (14), the value of the inputs for lower limb and SAAM is between 0 and 1, and the outputs are also the same.

According to Equation (1), through the singular value decomposition [25] of the normalized matrices *J*_01_′ and *J*_02_′ , which separately describe the kinematical performance without and with the assistive mechanism, there are

(16) $$\begin{array}{c} {J^{\prime }}_{0i_{0}}=U_{0i_{0}}\cdot S_{0i_{0}}\cdot V_{0i_{0}},\\ {J^{\prime }}_{0i_{0}}=A_{03}^{-1}\cdot J_{0i_{0}}\cdot A_{0i_{0}},\\ i_{0}=1,2. \end{array}$$

Some results, such as ME-al, MD-al, ME-saam, and MD-saam can be obtained from Equation (16). With the inputs, EV-al and EV-saam also can be obtained through Equations (6) and (8).

With the results above, in the following parts, it will validate and show MIP can be applicable on lower-limb assistive mechanism's design and optimization to evaluate the assistive feasibility and assistive ability.

### 4.2. MIP Study on Assistive Feasibility Validation

In this part, with some examples, MIP will be validated to be applicable to evaluate assistive feasibility for the lower-limb assistive mechanism. For a certain posture, satisfying assistive feasibility means the assistive mechanism can assist on all the directions. Hence, the first step is to validate assistive feasibility by considering one direction, and then, the validation extends to all the directions logically. Since MIP describes the relation between end-effectors' manipulability and velocities, the validation is based on end-effectors' maximum output velocities comparison and manipulability magnitude comparison.

At first, end-effectors' maximum output velocities on a certain direction for the lower limb and SAAM should be performed. As their end-effectors have the same two translational DOFs in the *X* − *Y* planar, *v*_1*x*_ and *v*_1*y*_ separately represents *X*-velocity component and *Y*-velocity component for end-effectors' velocity of the lower limb (EV-al); *v*_2*x*_ and *v*_2*y*_ also separately represent *X*-velocity component and *Y*-velocity component for end-effectors' velocity of SAAM (EV-saam). For lower limb and SAAM, while their end-effectors separately reaches the maximum velocity on direction $\vec{{\left(j_{i}\right)}_{w}}$, the constraint equations are

(17) $$\begin{array}{c} \left\{\begin{array}{c} \left|{\dot{\theta }}_{1}\right|={\left({\dot{\theta }}_{1}\right)}_{\max}\text{ or }\left|{\dot{\theta }}_{2}\right|={\left({\dot{\theta }}_{2}\right)}_{\max},\\ {\left[\begin{array}{cc} {\left(v_{1x}\right)}_{w} & {\left(v_{1y}\right)}_{w} \end{array}\right]}^{T}/\;/\vec{{\left(j_{i}\right)}_{w}}, \end{array}\right. \end{array}$$

(18) $$\begin{array}{c} \left\{\begin{array}{c} \left|{\dot{\theta }}_{3}\right|={\left({\dot{\theta }}_{3}\right)}_{\max}\text{ or }\left|{\dot{d}}_{4}\right|={\left({\dot{d}}_{4}\right)}_{\max},\\ {\left[\begin{array}{cc} {\left(v_{2x}\right)}_{w} & {\left(v_{2y}\right)}_{w} \end{array}\right]}^{T}/\;/\vec{{\left(j_{i}\right)}_{w}}, \end{array}\right. \end{array}$$

where in this paper, (*∗*)_*w*_ represents the results of case (*w*). In Equation (17), the constraint equations mean almost on all the directions, while lower limb's end-effector reaches its maximum velocity, only one master-active joint can reach its maximum velocity, where only on four directions both lower limb's master-active joints can reach maximum velocities simultaneously. Equation (18) also shows similar meaning for the SAAM.

Then for the manipulability, on direction $\vec{{\left(j_{i}\right)}_{w}}$, the lower limb's manipulability magnitude is

(19) $$\begin{array}{c} {\left(m_{1}\right)}_{w}=\sqrt{{\left(S_{01}^{2}\left(1\right)\right)}_{w}{\;\cos}^{2}{\left(\alpha_{1}\right)}_{w}+{\left(S_{01}^{2}\left(4\right)\right)}_{w}{\;\sin}^{2}{\left(\alpha_{1}\right)}_{w}},\\ {\left(\alpha_{1}\right)}_{w}=\langle \vec{{\left(j_{i}\right)}_{w}},{\left[\begin{array}{cc} U_{01}\left(1\right) & U_{01}\left(2\right) \end{array}\right]}_{w}^{T}\rangle . \end{array}$$

Here, 〈*∗*, *∗*〉 represents two vectors' intersection angle. And on this direction, the manipulability magnitude of SAAM is

(20) $$\begin{array}{c} {\left(m_{2}\right)}_{w}=\sqrt{{\left(S_{02}^{2}\left(1\right)\right)}_{w}{\;\cos}^{2}{\left(\alpha_{2}\right)}_{w}+{\left(S_{02}^{2}\left(4\right)\right)}_{w}{\;\sin}^{2}{\left(\alpha_{2}\right)}_{w}},\\ {\left(\alpha_{2}\right)}_{w}=\langle \vec{{\left(j_{i}\right)}_{w}},{\left[\begin{array}{cc} U_{02}\left(1\right) & U_{02}\left(2\right) \end{array}\right]}_{w}^{T}\rangle . \end{array}$$

In this paper, the items in a certain matrix *H* are defined as

(21) $$\begin{array}{c} H={\left[\begin{array}{cccc} H\left(1\right) & H\left(n+1\right) & \cdots & \vdots \\ \vdots & \vdots & \cdots & \vdots \\ H\left(n\right) & H\left(2n\right) & \cdots & H\left(n\times m\right) \end{array}\right]}_{n\times m}. \end{array}$$

Combined with Equations (17)–(20), on a certain direction, lower limb end-effector's and SAAM end-effector's maximum output velocities comparison is shown between Equations (17) and (18), and manipulability magnitude comparison is shown between Equations (19) and (20). Based on these two comparisons, the following examples will show MIP is applicable to evaluate assistive feasibility for the lower-limb assistive mechanism.

**Figure 4:**
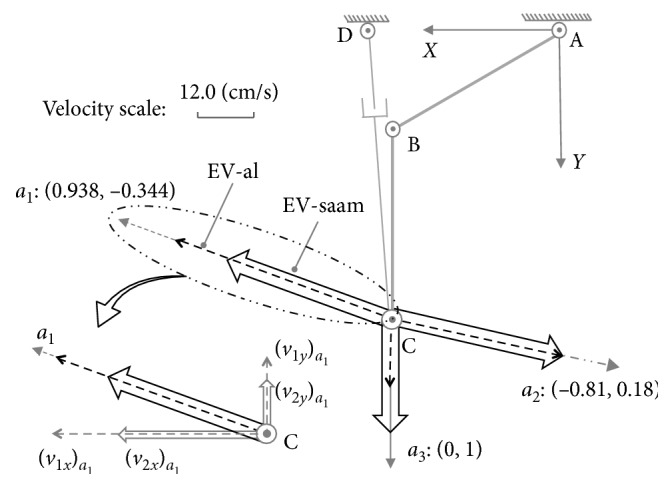
Maximum velocities comparison on different directions at posture (a): {*θ*_1_=*π*/6, *θ*_2_=*π*/3}.

In these examples, assume the parameters are set as *l*_1_=30(cm), *l*_2_=30(cm), ${\left({\dot{\theta }}_{1}\right)}_{\max}=\pi /5\left(\text{rad}/\text{s}\right)$, ${\left({\dot{\theta }}_{2}\right)}_{\max}=\pi /4\left(\text{cm}/\text{s}\right)$, *x*_0_=30(cm), *y*_0_=0(cm), ${\left({\dot{\theta }}_{3}\right)}_{\max}=\pi /4\left(\text{rad}/\text{s}\right)$, ${\left({\dot{d}}_{4}\right)}_{\max}=24\left(\text{cm}/\text{s}\right)$, and ${\left({\dot{x}}_{\text{C}}\right)}_{\max}={\left({\dot{y}}_{\text{C}}\right)}_{\max}=l_{1}\cdot {\left({\dot{\theta }}_{3}\right)}_{\max}+l_{2}\cdot {\left({\dot{\theta }}_{2}\right)}_{\max}=42.951\left(\text{cm}/\text{s}\right)$. As shown in Figure 4, for posture (a): {*θ*_1_=*π*/6, *θ*_2_=*π*/3}, through the singular value decomposition of (*J*′_01_)_*a*_ and (*J*′_02_)_*a*_, there are

(22) $$\begin{array}{c} {\left(U_{01}\right)}_{a}=\left[\begin{array}{cc} -0.938 & 0.344\\ 0.344 & 0.938 \end{array}\right],\\ {\left(S_{01}\right)}_{a}=\mathrm{diag}\left(0.92,0.23\right), \end{array}$$

(23) $$\begin{array}{c} {\left(U_{02}\right)}_{a}=\left[\begin{array}{cc} 0.996 & -0.09\\ 0.09 & 0.996 \end{array}\right],\\ {\left(S_{02}\right)}_{a}=\mathrm{diag}\left(0.84,0.57\right), \end{array}$$

where (*U*_01_)_*a*_ represents the MD-al, and (*S*_01_)_*a*_ represents the manipulability magnitude of assisted limb on each DOF. Similarly, (*U*_02_)_*a*_ represents the MD-saam, and (*S*_02_)_*a*_ is the manipulability magnitude of SAAM for each DOF.

Both the end-effectors of the assisted limb and SAAM move along direction *a*_1_ : [0.938, −0.344]. According to the constraint Equations (17) and (18), while the velocities of lower limb's joints are $\left\{{\dot{\theta }}_{1}=-{\left({\dot{\theta }}_{1}\right)}_{\max}=-\pi /5,{\dot{\theta }}_{2}=-0.541\right\}$, where master-active joint A reaches its maximum velocity, and in the SAAM, the velocities slave-active's joints are $\left\{{\dot{\theta }}_{3}=-{\left({\dot{\theta }}_{3}\right)}_{\max}=-\pi /4,{\dot{d}}_{4}=-9.531\right\}$, where ${\dot{\theta }}_{3}$ reaches its maximum velocity, the assisted limb's end-effector and SAAM's end-effector separately reaches their maximum velocities on direction*a*_1_

(24) $$\begin{array}{c} \left[\begin{array}{c} {\left(v_{1x}\right)}_{a_{1}}\\ {\left(v_{1y}\right)}_{a_{1}} \end{array}\right]={\left(J_{01}\right)}_{a}\cdot \left[\begin{array}{c} -{\left({\dot{\theta }}_{1}\right)}_{\max}\\ -0.541 \end{array}\right]=\left[\begin{array}{c} 44.511\\ -16.323 \end{array}\right], \end{array}$$

(25) $$\begin{array}{c} \left[\begin{array}{c} {\left(v_{2x}\right)}_{a_{1}}\\ {\left(v_{2y}\right)}_{a_{1}} \end{array}\right]={\left(J_{02}\right)}_{a}\cdot \left[\begin{array}{c} -{\left({\dot{\theta }}_{3}\right)}_{\max}\\ -9.531 \end{array}\right]=\left[\begin{array}{c} 34.494\\ -12.651 \end{array}\right], \end{array}$$

where the result of 44.511/(−16.323)=34.494/(−12.651)=0.938/(−0.344) shows end-effectors' velocities are along direction *a*_1_. In Figure 4, on direction *a*_1_, it also shows both the EV-al and EV-saam parallel with *a*_1_, where EV-al is the resultant velocity of *v*_1*x*_ and *v*_1*y*_, and EV-saam is the resultant velocity of *v*_2*x*_ and *v*_2*y*_. In Figure 4, end-effectors' maximum velocities comparison between Equations (24) and (25) shows that lower limb's end-effector can reach larger velocity than SAAM's end-effector in this case. From the aspect of manipulability, on direction *a*_1_, lower limb's manipulability magnitude is

(26) $$\begin{array}{c} {\left(m_{1}\right)}_{a_{1}}=\sqrt{{\left(S_{01}^{2}\left(1\right)\right)}_{a}{\;\cos}^{2}{\left(\alpha_{1}\right)}_{a_{1}}+{\left(S_{01}^{2}\left(4\right)\right)}_{a}{\;\sin}^{2}{\left(\alpha_{1}\right)}_{a_{1}}}\\ =0.92, \end{array}$$

where (*α*_1_)_*a*_1__=〈(0.938, −0.344), (−0.938, 0.344)〉=0. The manipulability magnitude of SAAM is

(27) $$\begin{array}{c} {\left(m_{2}\right)}_{a_{1}}=\sqrt{{\left(S_{02}^{2}\left(1\right)\right)}_{a}{\;\cos}^{2}{\left(\alpha_{2}\right)}_{a_{1}}+{\left(S_{02}^{2}\left(4\right)\right)}_{a}{\;\sin}^{2}{\left(\alpha_{2}\right)}_{a_{1}}}\\ =0.8, \end{array}$$

where (*α*_2_)_*a*_1__=〈(0.938, −0.344), (0.996, 0.09)〉=0.044. Thereby, there is (*m*_1_)_*a*_1__ > (*m*_2_)_*a*_1__. In this example, the comparison results mean that while SAAM's manipulability magnitude is smaller than lower limb's, SAAM end-effector's velocity is also smaller than lower limb end-effector's.

In the second example, on direction *a*_2_ : [−0.807, 0.184], while the velocities of lower limb's joints are: $\left\{{\dot{\theta }}_{1}=3.429,{\dot{\theta }}_{2}={\left({\dot{\theta }}_{2}\right)}_{\max}=\pi /4\right\}$, and the velocities of assistive mechanism's joints are: $\left\{{\dot{\theta }}_{3}={\left({\dot{\theta }}_{3}\right)}_{\max}=\pi /4,{\dot{d}}_{4}=4.839\right\}$, their end-effectors' maximum velocities on direction *a*_2_ are

(28) $$\begin{array}{c} \left[\begin{array}{c} {\left(v_{1x}\right)}_{a_{2}}\\ {\left(v_{1y}\right)}_{a_{2}} \end{array}\right]={\left(J_{01}\right)}_{a}\cdot \left[\begin{array}{c} 3.429\\ {\left({\dot{\theta }}_{2}\right)}_{\max} \end{array}\right]=\left[\begin{array}{c} -36.96\\ 8.442 \end{array}\right], \end{array}$$

(29) $$\begin{array}{c} \left[\begin{array}{c} {\left(v_{2x}\right)}_{a_{2}}\\ {\left(v_{2y}\right)}_{a_{2}} \end{array}\right]={\left(J_{02}\right)}_{a}\cdot \left[\begin{array}{c} {\left({\dot{\theta }}_{3}\right)}_{\max}\\ 4.839 \end{array}\right]=\left[\begin{array}{c} -36.96\\ 8.442 \end{array}\right]. \end{array}$$

The results in Equations (28) and (29) mean, just as shown in Figure 4, on direction *a*_2_, the end-effectors for the lower limb and SAAM can achieve the same maximum velocity. Their separate manipulability magnitude is (*m*_1_)_*a*_2__=(*m*_2_)_*a*_2__=0.816. In this example, it means while the manipulability magnitude for lower limb and SAAM is equal, the maximum output velocities on the certain direction are also equal.

The last example is on direction *a*_3_ : [0,1], where there are $\left\{{\dot{\theta }}_{1}=0.524,{\dot{\theta }}_{2}=-{\left({\dot{\theta }}_{2}\right)}_{\max}=-\pi /4\right\}$, and $\left\{{\dot{\theta }}_{3}=-0.047,{\dot{d}}_{4}={\left({\dot{d}}_{4}\right)}_{\max}=24\right\}$. Therefore, we can have

(30) $$\begin{array}{c} \left[\begin{array}{c} {\left(v_{1x}\right)}_{a_{3}}\\ {\left(v_{1y}\right)}_{a_{3}} \end{array}\right]={\left(J_{01}\right)}_{a}\cdot \left[\begin{array}{c} 0.524\\ -{\left({\dot{\theta }}_{2}\right)}_{\max} \end{array}\right]=\left[\begin{array}{c} 0\\ 13.59 \end{array}\right],\\ \left[\begin{array}{c} {\left(v_{2x}\right)}_{a_{3}}\\ {\left(v_{2y}\right)}_{a_{3}} \end{array}\right]={\left(J_{02}\right)}_{a}\cdot \left[\begin{array}{c} -0.047\\ {\left({\dot{d}}_{4}\right)}_{\max} \end{array}\right]=\left[\begin{array}{c} 0\\ 24.09 \end{array}\right]. \end{array}$$

Surely, SAAM can act quicker than the lower limb just as shown in Figure 4 on direction *a*_3_. Their manipulability magnitude on this direction is (*m*_1_)_*a*_3__=0.383 and (*m*_2_)_*a*_3__=0.573. There is (*m*_1_)_*a*_3__ < (*m*_2_)_*a*_3__. Thereby, in this example, while SAAM end-effector's manipulability magnitude is larger than lower limb end-effector's, SAAM end-effector's velocity is also larger than lower limb end-effector's. Therefore, it can ensure the assistive mechanism shadows and assist lower limb's movement on this direction.

Through these three examples, the comparison results verify that on a certain direction, if SAAM end-effector's manipulability magnitude is equal to, larger than, or smaller than lower limb end-effector's, SAAM end-effector's maximum velocities are equal to, larger than, or smaller than lower limb end-effector's. It shows the end-effectors' manipulability magnitude comparison is directly related to end-effectors' maximum velocities comparison.

Further, as well known, the manipulability magnitude and velocity for mechanism's end-effector have the same change tendency, where the manipulability magnitude becomes smaller or larger on certain directions, and the maximum velocity also decreases or increases on certain directions. Thereby, we can extend the comparison results from one direction to all directions logically: if SAAM's manipulability magnitude is larger than lower limb's on all the directions, assistive mechanism satisfies the assistive feasibility since it can shadow lower-limb end-effector's velocity on all the directions; if SAAM's manipulability magnitude is smaller than lower limb's on all the directions, assistive mechanism cannot satisfy the assistive feasibility since it cannot shadow lower-limb end-effector's velocity at all; if SAAM's manipulability magnitude is larger than, equal to, or smaller than lower limb's on part of all the directions, the assistive mechanism may or may not shadow lower limb's velocity on part of all the directions, where in this case, the assistive mechanism also cannot satisfy assistive feasibility.

The above can be concluded as follows: when assisted limb's manipulability magnitude is smaller than or equal to SAAM's on all directions, in other words, when ME-al is whole inclusive by ME-saam, the assistive mechanism can shadow the assisted limb's movements well to satisfy assistive feasibility and be used to provide assistance on all the directions. This conclusion accords to the assistive feasibility evaluation criterion shown in the definition of MIP; therefore, MIP is proved to be applicable to evaluate lower-limb assistive mechanism's assistive feasibility.

In order to evaluate assistive feasibility for assistive mechanism, manipulability magnitude comparison with one direction by one direction is almost inefficient. Therefore, it is necessary to find an inclusive judgement algorithm which can judge the inclusive case on the whole directions quickly.

### 4.3. Inclusive Judgement Algorithm

Since the manipulability ellipsoid is used to represent the manipulability magnitude on the whole directions, we can compare the ME-al and ME-saam in terms of geometry to obtain the manipulability magnitude comparison results on all the directions. Through the inclusive judgement algorithm, we can judge whether the assistive mechanism can satisfy assistive feasibility or not.

At first, according to the first two concerns shown in the problem of assistive feasibility, lower limb end-effector's and SAAM end-effector's DOF spaces should be in the same space. It means their manipulability ellipsoids should be also in the same space. For the lower limb assistance, the manipulability ellipsoids of lower limb and SAAM both degenerate to ellipses. That whether these two ellipses are in the same space or not can be judged by their major and minor axes' normal lines: if these two normal lines are parallel to each other, these two ellipses are in the same space; otherwise they are not in the same space.

**Figure 5:**
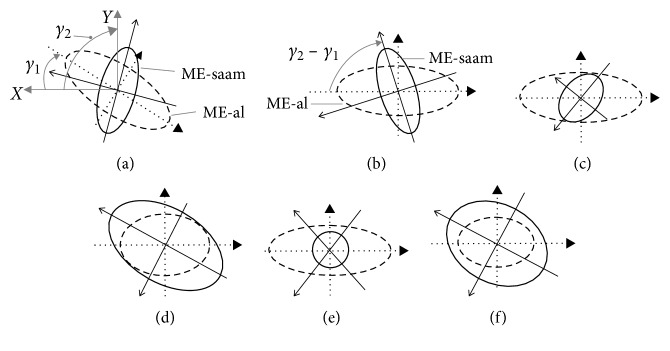
Inclusive cases between ME-al and ME-saam. (a) Initial, (b) after rotation, (c) internally tangent, (d) externally tangent, (e) internally inclusive, (f) externally inclusive.

Then, the third concern can be judged by the intersection of these two ellipses. Assuming the intersection angles between these two ellipses and axis *X* are *γ*_1_ *and* *γ*_2_, just as shown in Figure 5(a), the expressions for the manipulability ellipses of lower limb and SAAM are

(31) $$\begin{array}{c} \frac{{\left(\cos\gamma_{1}\cdot x+\sin\gamma_{1}\cdot y\right)}^{2}}{S_{1}^{2}\left(1\right)}+\frac{{\left(-\sin\gamma_{1}\cdot x+\cos\gamma_{1}\cdot y\right)}^{2}}{S_{1}^{2}\left(4\right)}=1,\\ \frac{{\left(\cos\gamma_{2}\cdot x+\sin\gamma_{2}\cdot y\right)}^{2}}{S_{2}^{2}\left(1\right)}+\frac{{\left(-\sin\gamma_{2}\cdot x+\cos\gamma_{2}\cdot y\right)}^{2}}{S_{2}^{2}\left(4\right)}=1. \end{array}$$

For the sake of computation, make the same rotations for these two ellipses. Then, Figure 5(b) is the rotation results of Figure 5(a), where the major and minor axes of MD-al align with axes *X* and *Y*. There are

(32) $$\begin{array}{c} \frac{x^{2}}{S_{1}^{2}\left(1\right)}+\frac{y^{2}}{S_{1}^{2}\left(4\right)}=1, \end{array}$$

(33) $$\begin{array}{c} \frac{{\left(\cos\gamma \cdot x+\sin\gamma \cdot y\right)}^{2}}{S_{2}^{2}\left(1\right)}+\frac{{\left(-\sin\gamma \cdot x+\cos\gamma \cdot y\right)}^{2}}{S_{2}^{2}\left(4\right)}=1, \end{array}$$

where *γ*=*γ*_2_ − *γ*_1_.

Since the ME-al and ME-saam are both odd symmetry about axis *X* as shown in Figure 5(b), we can only discuss the case of *y* ≥ 0 to judge their intersection or inclusive cases,

(34) $$\begin{array}{c} y\geq 0. \end{array}$$

By solving the set of Equations (32)–(34) which are quadratic equations about {*x*, *y*} to obtain real roots, there exist three cases: two roots, sole root, and no root. The cases of roots are directly related to the intersection or inclusive cases.

For example, just as shown in Figure 5(b), ME-al is part inclusive by ME-saam with two different points of intersection, where in this case, the set of equations has two roots. When the set of equations has sole root, the inclusive cases are internally tangent (Figure 5(c)) or externally tangent (Figure 5(d)), where we can separate them by considering their major and minor axes extremities distribution. If the set of equations has no root, it means ME-al and ME-saam have no point of intersection. In this case, it also has two types which can also be separated by considering their major and minor axes extremities distribution: internally inclusive (Figure 5(e)) and externally inclusive (Figure 5(f)).

The inclusive cases between ME-al and ME-saam are considered to be divided into three types: part inclusive case (Figures 5(a) and 5(b)), no inclusive case (Figures 5(c) and 5(e)), and whole inclusive case (Figures 5(d) and 5(f)). With this inclusive judgement algorithm, the inclusive case between ME-al and ME-saam can be obtained quickly.

In the following part, through the study on kinds of inclusive cases, we can understand which case is as our expected. Moreover, by the study on all kinds of inclusive cases, we can further understand MIP is applicable to evaluate assistive feasibility for lower-limb assistive mechanism, where it is also needed to check whether this inclusive judgement algorithm is applicable to judge inclusive cases or not.

### 4.4. Cases of Part Inclusive, No Inclusive, and Whole Inclusive

Through comparing the ME-al and ME-saam with inclusive judgement algorithm, for a certain assistive mechanism, at different postures there may exist three cases: part inclusive case, no inclusive case, and whole inclusive case.

#### 4.4.1. Part Inclusive Case

**Figure 6:**
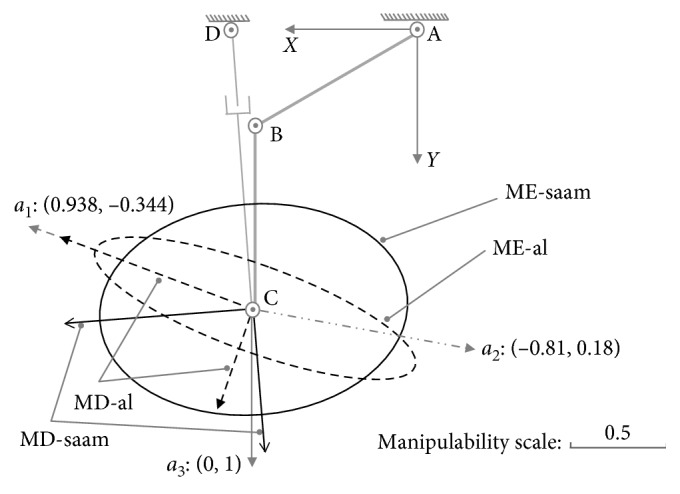
Part inclusive case at posture (a): {*θ*_1_=*π*/6, *θ*_2_=*π*/3}.

For posture (a): {*θ*_1_=*π*/6, *θ*_2_=*π*/3}, combined with Equation (22), we can draw the ME-al (denoted with dashed ellipse) with (*U*_01_)_*a*_ and (*S*_01_)_*a*_ to represent the manipulability of lower limb just as shown in Figure 6. Similarly, the ME-saam (denoted with solid ellipse) is generated with (*U*_02_)_*a*_ and (*S*_02_)_*a*_ to represent the manipulability of SAAM. In Figure 6, ME-al is part inclusive by the ME-saam, where by the inclusive judgement algorithm, the set of Equations (32)–(34) also shows that it has two different roots in this case.

With MIP, for the part inclusive case, there must be the conclusion: in different directions, SAAM end-effector's manipulability magnitude may be smaller than, equal to, or larger than lower-limb end-effector's; correspondingly, on these different directions, SAAM end-effector's maximum velocity is smaller than, equal to, or larger than lower limb's. In Figure 4, the maximum velocities comparison results on different directions certify this conclusion.

As the discussion before (Figure 4), in direction *a*_1_, SAAM cannot catch lower limb's movement; on direction *a*_2_, their end-effectors' maximum velocities are equal; on direction *a*_3_, SAAM end-effector's maximum velocity is larger than lower-limb end-effector's.

The above means, for the case of part inclusive, in some directions, the assistive mechanism cannot shadow lower limb's movements to assist, but on the rest of the directions it can.

#### 4.4.2. No Inclusive Case

At posture (b): {*θ*_1_=−1.1, *θ*_2_=2.02}, there are

(35) $$\begin{array}{c} {\left(U_{01}\right)}_{b}=\left[\begin{array}{cc} 0.52 & 0.854\\ 0.854 & -0.52 \end{array}\right],\\ {\left(S_{01}\right)}_{b}=\mathrm{diag}\left(0.64,0.35\right),\\ {\left(U_{02}\right)}_{b}=\left[\begin{array}{cc} 0.517 & 0.856\\ 0.856 & -0.517 \end{array}\right],\\ {\left(S_{02}\right)}_{b}=\mathrm{diag}\left(0.57,0.06\right). \end{array}$$

**Figure 7:**
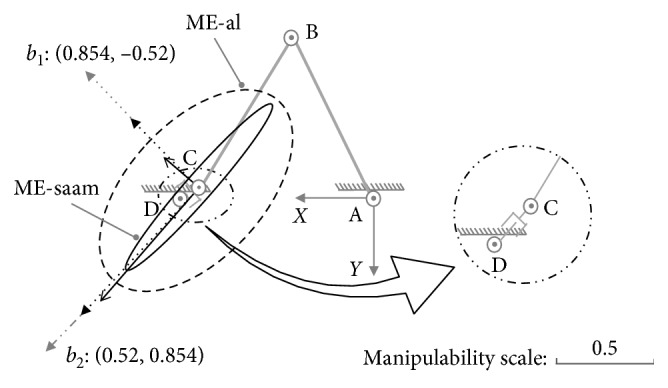
No inclusive case at posture (b): {*θ*_1_=−1.1, *θ*_2_=2.02}.

**Figure 8:**
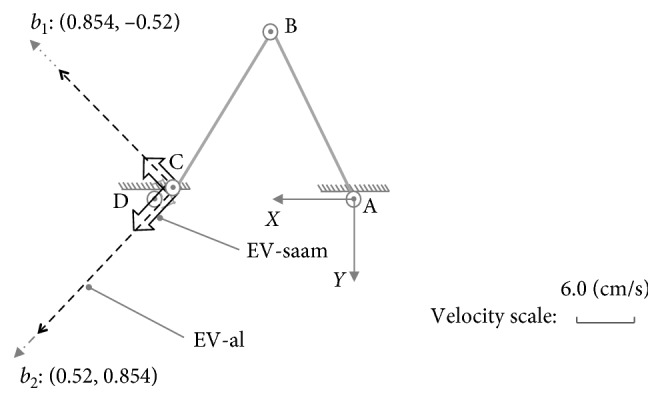
Maximum velocity comparison on different directions for part inclusive case at posture (b).

In this case, with the inclusive judgement algorithm, the set of Equations (32)–(34) shows it has no real roots, and the major and minor axes extremities of ME-al are at the outer of ME-saam. Hence, this posture belongs to no inclusive case just as shown in Figure 7. According to MIP, in this case, on all the directions, SAAM's manipulability magnitude is smaller than lower limb's (Figure 7); correspondingly, SAAM end-effector's maximum velocities are also smaller than lower limb end-effector's. This conclusion can be certified by the maximum velocities comparison results shown in Figure 8.

As shown in Figure 8, while lower limb and SAAM move along direction *b*_1_ : [0.854, −0.52], there are

(36) $$\begin{array}{c} \left[\begin{array}{c} {\left(v_{1x}\right)}_{b_{1}}\\ {\left(v_{1y}\right)}_{b_{1}} \end{array}\right]={\left(J_{01}\right)}_{b}\cdot \left[\begin{array}{c} 0.085\\ -{\left({\dot{\theta }}_{2}\right)}_{\max} \end{array}\right]=\left[\begin{array}{c} 18.99\\ -5.25 \end{array}\right], \end{array}$$

(37) $$\begin{array}{c} \left[\begin{array}{c} {\left(v_{2x}\right)}_{b_{1}}\\ {\left(v_{2y}\right)}_{b_{1}} \end{array}\right]={\left(J_{02}\right)}_{b}\cdot \left[\begin{array}{c} {\left({\dot{\theta }}_{3}\right)}_{\max}\\ 5.25 \end{array}\right]=\left[\begin{array}{c} 5.01\\ -3.06 \end{array}\right]. \end{array}$$

In Equations (36) and (37), lower limb end-effector's velocity is larger. On direction *b*_2_ : [0.52, 0.854], there are

(38) $$\begin{array}{c} \left[\begin{array}{c} {\left(v_{1x}\right)}_{b_{2}}\\ {\left(v_{1y}\right)}_{b_{2}} \end{array}\right]={\left(J_{01}\right)}_{b}\cdot \left[\begin{array}{c} {\left({\dot{\theta }}_{1}\right)}_{\max}\\ -0.3 \end{array}\right]=\left[\begin{array}{c} 8.88\\ 14.58 \end{array}\right], \end{array}$$

(39) $$\begin{array}{c} \left[\begin{array}{c} {\left(v_{2x}\right)}_{b_{2}}\\ {\left(v_{2y}\right)}_{b_{2}} \end{array}\right]={\left(J_{02}\right)}_{b}\cdot \left[\begin{array}{c} {\left({\dot{\theta }}_{3}\right)}_{\max}\\ -1.35 \end{array}\right]=\left[\begin{array}{c} 1.56\\ 2.55 \end{array}\right]. \end{array}$$

The results in Equations (38) and (39) also show SAAM cannot catch lower limb end-effector's velocities.

Therefore, in the case of no inclusive, SAAM cannot catch lower-limb end-effector's movements on all the directions. Moreover, since SAAM end-effector's velocities are lower than lower limb end-effector's, it will drag lower limb's movements. Hence, in the case of no inclusive, the assistive mechanism cannot assist the lower limb at all.

#### 4.4.3. Whole Inclusive Case

In order to assist the lower limb on all the directions, part inclusive case and no inclusive case must be avoided. For posture (c): {*θ*_1_=*π*/5, *θ*_2_=3.02}, there are

(40) $$\begin{array}{c} {\left(U_{01}\right)}_{c}=\left[\begin{array}{cc} -0.615 & 0.788\\ 0.788 & 0.615 \end{array}\right],\\ {\left(S_{01}\right)}_{c}=\mathrm{diag}\left(0.556,0.005\right),\\ {\left(U_{02}\right)}_{c}=\left[\begin{array}{cc} -0.084 & 0.996\\ 0.996 & 0.084 \end{array}\right],\\ {\left(S_{02}\right)}_{c}=\mathrm{diag}\left(0.603,0.566\right). \end{array}$$

**Figure 9:**
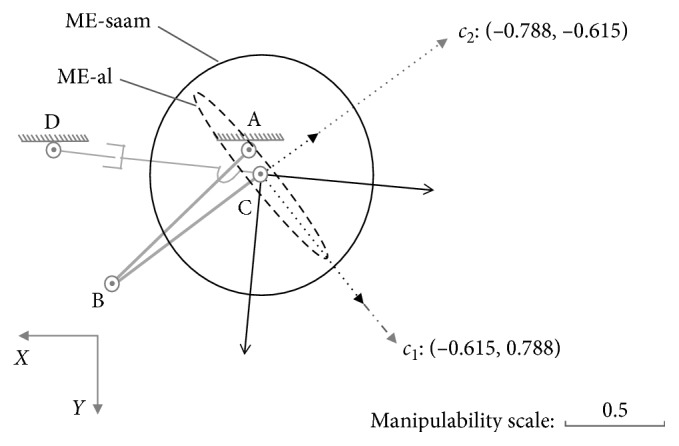
Whole inclusive case at posture (c): {*θ*_1_=*π*/5, *θ*_2_=3.02}.

For this posture, by the inclusive judgement algorithm, the set of Equations (32)–(34) shows it has no real roots, and the major and minor axes extremities of ME-al are at the inner of ME-saam. Therefore, ME-al is whole inclusive by ME-saam just as shown in Figure 9. According to MIP, in the case of whole inclusive, SAAM end-effector's velocities are larger than lower-limb end-effector's on all the directions.

**Figure 10:**
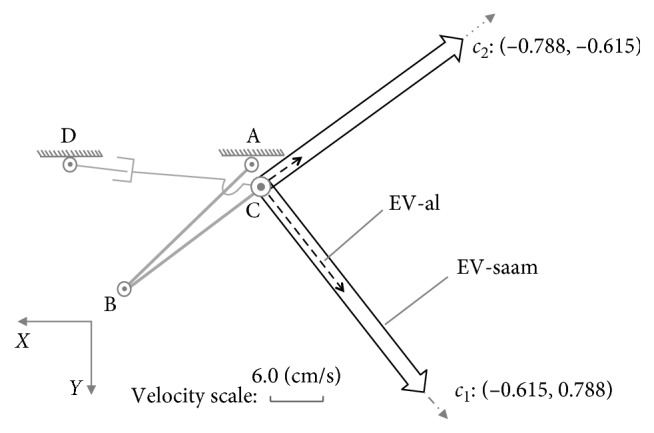
Maximum velocity comparison on different directions for whole inclusive case at posture (c).

In this case, as shown in Figure 10, on direction *c*_1_ : [−0.615, 0.788], there are

(41) $$\begin{array}{c} \left[\begin{array}{c} {\left(v_{1x}\right)}_{c_{1}}\\ {\left(v_{1y}\right)}_{c_{1}} \end{array}\right]={\left(J_{01}\right)}_{c}\cdot \left[\begin{array}{c} {\left({\dot{\theta }}_{1}\right)}_{\max}\\ -0.49 \end{array}\right]=\left[\begin{array}{c} -9.06\\ 11.61 \end{array}\right], \end{array}$$

(42) $$\begin{array}{c} \left[\begin{array}{c} {\left(v_{2x}\right)}_{c_{1}}\\ {\left(v_{2y}\right)}_{c_{1}} \end{array}\right]={\left(J_{02}\right)}_{c}\cdot \left[\begin{array}{c} {\left({\dot{\theta }}_{3}\right)}_{\max}\\ -1.62 \end{array}\right]=\left[\begin{array}{c} -18.39\\ 23.55 \end{array}\right]. \end{array}$$

And on direction *c*_2_ : [−0.788, −0.615], we have

(43) $$\begin{array}{c} \left[\begin{array}{c} {\left(v_{1x}\right)}_{c_{2}}\\ {\left(v_{1y}\right)}_{c_{2}} \end{array}\right]={\left(J_{01}\right)}_{c}\cdot \left[\begin{array}{c} {\left({\dot{\theta }}_{1}\right)}_{\max}\\ 0.007 \end{array}\right]=\left[\begin{array}{c} -1.83\\ -1.41 \end{array}\right], \end{array}$$

(44) $$\begin{array}{c} \left[\begin{array}{c} {\left(v_{2x}\right)}_{c_{2}}\\ {\left(v_{2y}\right)}_{c_{2}} \end{array}\right]={\left(J_{02}\right)}_{c}\cdot \left[\begin{array}{c} 0.48\\ -{\left({\dot{d}}_{4}\right)}_{\max} \end{array}\right]=\left[\begin{array}{c} -22.44\\ -17.49 \end{array}\right]. \end{array}$$

From the results above shown in Equations (41)–(44), on these directions, SAAM's end-effector can act quicker than lower limb's end-effector. Thereby, in the case of whole inclusive, the assistive mechanism can shadow lower limb's movements well to provide power assisting for lower limb on all the directions.

Through the study on these three kinds of inclusive cases, it shows the inclusive case can be confirmed with the inclusive judgement algorithm effectively. It also shows the MIP is quite useful on evaluating assistive feasibility for lower limb assistive mechanism. In order to provide assistance on all the directions, the ME-al must be whole inclusive by ME-saam. Otherwise, when the ME-saam cannot cover ME-al on some directions, it means the assistive mechanism cannot provide power assisting on these directions, and even worse, human will feel difficulty increased on these directions. Hence, only the case of whole inclusive ensures the assistive mechanism can be used to assist lower limb's movements on all the directions.

Therefore, the problem of evaluating assistive feasibility in fact is converted to the following: with the inclusive judgement algorithm, comparing the ME-saam and ME-al to ascertain assistive mechanism's assistive feasibility. Through the study on these parts, MIP is validated to be able to evaluate assistive feasibility for lower-limb assistive mechanism.

Different from the problem of evaluating assistive feasibility whose answer is about being able to assist or being not able to assist these two aspects, the problem of evaluating effect is about that how the assistive mechanism assist lower limb, where the evaluation for assistive effect is shown in Section 4. In the next part, we will analyze and discuss the influence factors for assistive feasibility and assistive effect.

### 4.5. Study on the Influence Factors of MIP

As the discussions above, for a certain assistive mechanism, at different postures, the inclusive cases may be different. Our purpose is the assistive mechanism can satisfy assistive feasibility and realize better assistive effect in the whole expected workspace. Thereby, it is necessary to analyze and discuss which factors will have the influence on MIP, and then, we can develop the assistance for the assistive mechanism, where the influence on MIP also means the influence on inclusive results or assistive results.

The definition of MIP is based on manipulability which can be obtained through kinematical Jacobian. Hence, the MIP is related to mechanical structure, sizes, types of joints, actuators' maximum velocities, assistive mechanism's connected and fixed locations, and so on. In this paper, we focus on the influence factors of actuators' maximum velocities and assistive mechanism's fixed location.

#### 4.5.1. Influence Factor of Actuators' Maximum Velocities

In this part, we will discuss the factor of actuators' maximum velocities, where in this case, only actuators' maximum velocities change. At posture (d), which is the same as posture (a), for the slave-active joints, the maximum velocity of ${\left({\dot{d}}_{4}\right)}_{\max}$ decreases from 24 (cm/s) to 8.28 (cm/s), and ${\left({\dot{\theta }}_{3}\right)}_{\max}$ decreases from *π*/4(rad/s) to 0.18 (rad/s). Then, SAAM's manipulability becomes

(45) $$\begin{array}{c} {\left(U_{02}\right)}_{d}=\left[\begin{array}{cc} 0.996 & -0.09\\ 0.09 & 0.996 \end{array}\right],\\ {\left(S_{02}\right)}_{d}=\mathrm{diag}\left(0.2,0.2\right), \end{array}$$

while the manipulability of lower limb remains the same as posture (a) shown in Equation (22).

**Figure 11:**
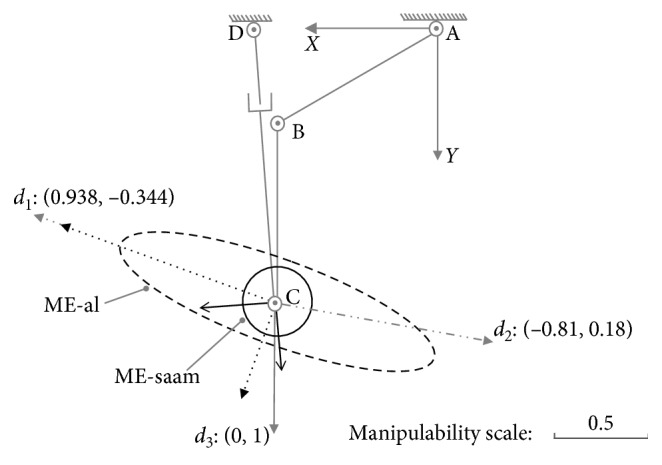
Influence with the change of actuators' maximum velocities at posture (d)(=(a)): {*θ*_1_=*π*/6, *θ*_2_=*π*/3}.

Compared to Figure 6, the inclusive case becomes from part inclusive case to no inclusive case just as shown in Figure 11. It shows the change of actuators' maximum velocities will have the influence on the MIP.

**Figure 12:**
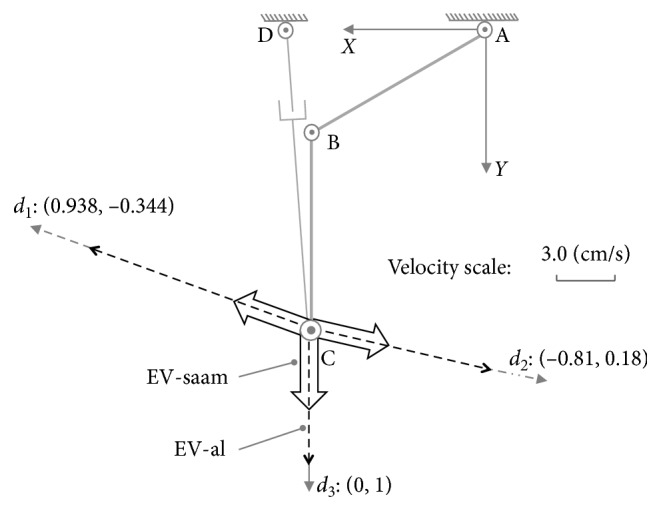
Maximum velocity comparison on different directions at posture (d)(=(a)) while actuators' maximum velocities change.

The change will also have the influence on end-effector's velocity. Compared to Figure 4, in Figure 12, besides directions *d*_1_(=*a*_1_), *d*_2_(=*a*_2_), and *d*_3_(=*a*_3_), SAAM end-effector's velocities also decrease on the other directions.

In this case, if the maximum velocities of actuators change, inclusive results will also change. In addition, in this case, driven by the assistive mechanism, the ME-saam in Figure 11 becomes a circle which possesses good features on symmetry and isotropy, where Figure 12 also shows SAAM end-effector's velocities on each direction are equal. However, in this case the assistive mechanism cannot assist at all. Therefore, during the design and optimization for assistive mechanism, only considering the symmetry and isotropy is not enough. For assistive mechanism, we must also consider the basic feature for assistive mechanism at first: assistive feasibility.

#### 4.5.2. Influence Factor of Fixed Location

**Figure 13:**
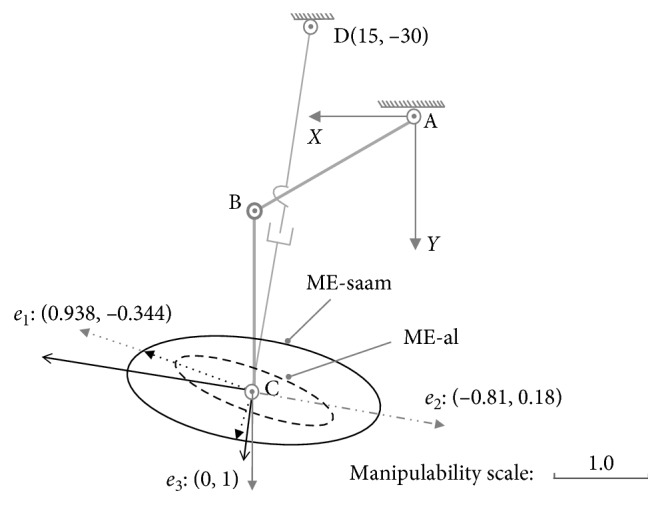
Influence with the change of fixed location at posture (e)(=(a)): {*θ*_1_=*π*/6, *θ*_2_=*π*/3}.

For assistive mechanism's fixed location D(*x*_0_, *y*_0_), in Figure 13, it shows only D changes from (30, 0) to (15, −30) with the same actuators, where lower limb is at posture (e) which is also the same as posture (a). In this case, the manipulability magnitude of SAAM becomes (*S*_02_)_*e*_=diag(1.40, 0.57). Compared to (*S*_02_)_*a*_=diag(0,84,0.57), the manipulability magnitude of revolute DOF is enhanced. Then compared to Figure 6, the inclusive case becomes from part inclusive case to whole inclusive case.

**Figure 14:**
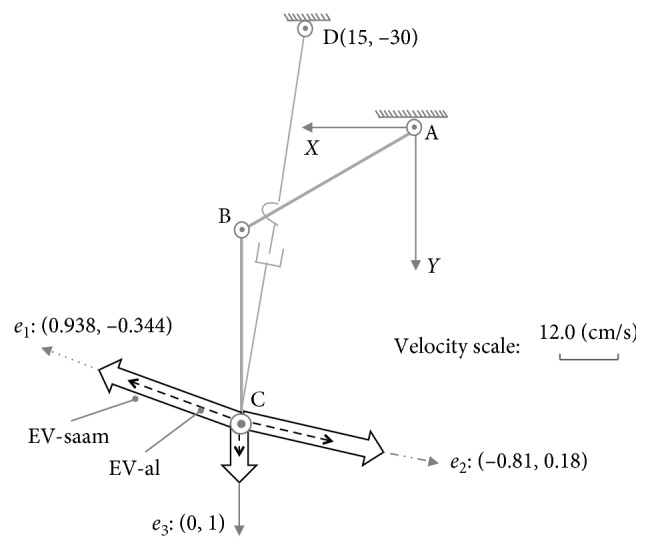
Maximum velocity comparison on different directions at posture (e)(=(a)) while fixed location changes.

In this case, just as shown in Figure 14, SAAM end-effector's velocities are also enhanced. Compared to Figure 4, in Figure 14, SAAM end-effector's velocities becomes larger than lower limb end-effector's on all the directions. It shows that changing fixed location will also have influence on the MIP.

From the discussions on the MIP influence factors, for the same posture, actuators' maximum velocities change and fixed location change will have influence on the inclusive case or assistive feasibility. Moreover, the changes of these factors will also have influence on the assistive effect. For example, at posture (a), the intersection angle between the major axes of MD-al and MD-saam in Figure 6 is 0.436(rad). While the fixed location changes, at the same posture (e) just as shown in Figure 13, the intersection angle decreases to 0.192(rad), where this development means after changing parameters, the MD-saam can align with MD-al much better to realize higher assistive efficiency. Meanwhile, compared to Figure 6, the volume of ME-saam in Figure 13 becomes much larger showing its assistive ability is enhanced. The change of assistive isotropy can be easily seen by comparing the ME-saam in Figures 6 and 11.

Hence, based on MIP, the optimization by considering the influence factors is helpful to make the assistive mechanism fit MIP. In other words, optimizing these parameters can relieve the problem of assistive feasibility, where changing the parameters can make no inclusive case or part inclusive case become whole inclusive case. And optimizing the parameters also makes sense for developing the assistive effect.

Through the studies on MIP in this section, it is certified that MIP can be used as the evaluation criterion for evaluating assistive feasibility and assistive effect for lower-limb assistive mechanism design and optimization. In order to realize better assistance in the whole expected workspace, the optimization algorithm based on MIP is needed.

## 5. Conclusions

In this paper, by considering satisfying assistive feasibility and realizing better assistive effect, the Manipulability Inclusive Principle (MIP) evaluation criterion for assistive mechanism design and optimization is proposed. This principle can be applicable on the assistive mechanisms which belong to the type where the human is acting actively, and simultaneously the assistive mechanism is matching human's moving intention.

In order to ensure that the expected assistive mechanism satisfies assistive feasibility, the manipulability ellipsoid of the assisted limb must be whole inclusive by the slave-active-assistive mechanism's manipulability ellipsoid. The inclusive cases can be confirmed with the inclusive judgement algorithm. The MIP also shows that different parameters, such as actuators' maximum velocities, fixed and connected locations, types of joints, mechanical structure, and sizes, will have the influence on the assistive results.

The manipulability inclusive principle can also be applied on other various assistive mechanisms. Through building the model for the assisted limb and slave-active-assistive mechanism and then comparing their manipulability, manipulability inclusive principle is useful for evaluating assistive mechanism's assistive feasibility and assistive effect during design and optimization.

## Data Availability

The data used to support the findings of this study are included within the article.
